# Early Bortezomib Therapy for Refractory Anti-NMDA Receptor Encephalitis

**DOI:** 10.3389/fneur.2020.00188

**Published:** 2020-03-27

**Authors:** Marion T. Turnbull, Jason L. Siegel, Tara L. Becker, Alana J. Stephens, A. Sebastian Lopez-Chiriboga, William D. Freeman

**Affiliations:** ^1^Department of Neurology, Mayo Clinic, Jacksonville, FL, United States; ^2^Department of Critical Care Medicine, Mayo Clinic, Jacksonville, FL, United States; ^3^Department of Neurologic Surgery, Mayo Clinic, Jacksonville, FL, United States

**Keywords:** anti-NMDA receptor encephalitis, bortezomib, proteosome inhibitor, autoimmune disease, case report

## Abstract

**Introduction:** Anti-*N*-methyl-D-aspartate (NMDA) receptor encephalitis is an increasingly recognized form of immune-mediated encephalitis. Here we present a case that represents the shortest hospitalization-to-bortezomib treatment timeline (42 days), and we believe that this is reflected in the patient's outcome with complete independence within a short timeframe.

**Case Report:** We describe a case of anti-NMDA receptor encephalitis in an 18-year-old African American female presenting with progressive, medically refractory disease. Despite two rounds of high-dose intravenous steroids, plasma exchange, immunoglobulin administration, and rituximab for B-cell depletion, the patient failed to respond by hospital day 42 and received off-label use of the proteasome inhibitor bortezomib. During the 15 days after the bortezomib administration, the patient showed dramatic neurologic recovery that allowed her transfer out of the intensive care unit. At follow-up after 1-month, the patient reported feeling normal cognitively and showed dramatic improvement in cognitive scores.

**Conclusion:** This case and literature review provide preliminary evidence that early treatment of anti-NMDA receptor encephalitis with the proteasome inhibitor bortezomib appears safe and tolerable. However, randomized trials are needed to show the efficacy and the long-term benefit.

## Introduction

Anti-*N*-methyl-D-aspartate (NMDA) receptor encephalitis is an antibody-mediated disorder presenting with psychiatric symptoms, movement disorders, seizures, autonomic instability, and sometimes profound alteration of consciousness with potential life-threatening complications (1). It can occur as a paraneoplastic condition—first identified in young women with ovarian teratomas (2)—a primary autoimmune, or a parainfectious condition, the latter being commonly triggered by a prior herpes simplex virus infection (3, 4).

Anti-NMDA receptor encephalitis is an immunotherapy responsive disorder (5). First-line treatment includes immunotherapy agents such as steroids, plasma exchange procedures (PLEX), and intravenous immunoglobulin (IVIg), and the second-line therapy includes B-cell depleting agents such as rituximab (6). Practical guidelines for differential diagnoses have been published (7); however, there is no consensus on treatment (6). To date there have been no prospective clinical trials to evaluate the treatment options and existing evidence is graded as class IV (8). Moreover, a large subset of patients do not improve following first-line therapy (9), highlighting the need for disease-stage-specific therapeutics and new controlled, randomized clinical trials.

Here we report the successful use of the proteasome inhibitor, bortezomib, in a case of anti-NMDA receptor encephalitis. Bortezomib targets antibody-secreting plasma cells resistant to B-cell depleting strategies (10, 11) and is a potential second-line therapeutic strategy for anti-NMDA receptor encephalitis (11–15). This case represents the shortest hospitalization-to-bortezomib treatment timeline (42 days), and we believe that this is reflected in the patient's outcome with complete independence within a short timeframe. We propose that bortezomib warrants the need for further investigation in clinical trials.

## Case Report

**Figure 1:**
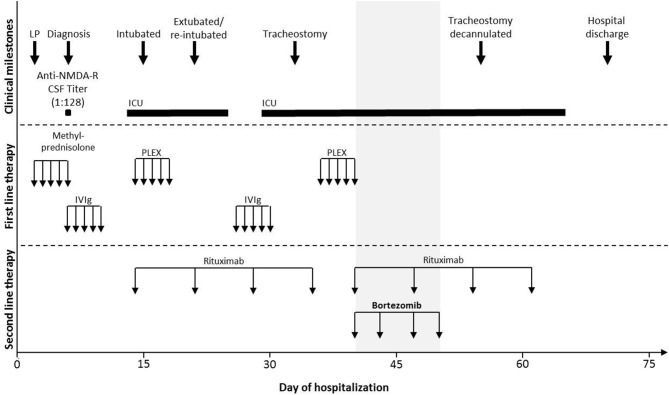
Clinical milestones and treatment course of the illustrative case. Schematic representation of the clinical course and the treatment divided into three vertical categories (dotted line): clinical milestones, first-line immunotherapy, and second-line immunotherapy. The X-axis indicates the day of hospitalization, and the gray area indicates the time of bortezomib administration. Anti-NMDA-R, anti-N-methyl-D-aspartate receptor; CSF, cerebrospinal fluid; ICU, intensive care unit; IVIg, intravenous immunoglobulin; LP, lumbar puncture; PLEX, plasma exchange.

An 18-year-old African American female with no previous medical history presented with seizure-like activity in the upper extremities at a local emergency department. After a negative head computed tomography (CT) scan and treatment with antiepileptic drugs, she was discharged from the hospital. Over the next several days, she developed progressive mood changes with emotional lability, child-like behavior, confusion, and slow mentation and was admitted to our hospital. The remainder of her complete neurological examination was normal. The head CT was again negative and magnetic resonance imaging (MRI) with contrast identified no structural lesions. During the next 3 days, behavioral issues progressed with agitation and aggressive behavior. Initial cerebrospinal fluid (CSF) studies revealed elevated white blood cell count with lymphocytic predominance (WBC 21, lymphocytes 92%, RBC <1, protein 16, cells counted 100, glucose 81), and she was started on 1,000 mg methylprednisolone for 5 days (Figure 1).

On days 3 and 4, the patient developed episodes of lip smacking and right hand tremors. Electroencephalograms revealed focal seizures arising from the left temporal lobe, and the patient was started on levetiracetam at up to 1,000 mg twice daily, but it was stopped due to the concern that it was contributing to her mental status issues. She was then put on fosphenytoin at 100 mg twice daily and lacosamide at 100 mg twice daily, but she continued to have left temporal focal seizures. Ultimately, she needed sedation due to agitation, requiring high doses of midazolam (30 mg/h) and propofol (100 mcg/kg/h), which stopped the seizures in addition to sedating her. On this regimen, she had breakthrough periods of attempting to get out of bed, sitting up, and near self-extubation; therefore, dexmedetomidine (max 2.0 mcg/kg/h) and fentanyl (max 200 mcg/h) were added. On day 6, neural autoantibody tested positive in serum for the NMDA receptor by cell-based assay, and in CSF (titer of 1:128), confirming the diagnosis of anti-NMDA receptor encephalitis. She was then started on a 5-day course of IVIg. At that time, the examination revealed a gaze-evoked nystagmus, ataxia, and dysmetria. Oncologic evaluation, including CT of the chest, the abdomen, and the pelvis with and without contrast, full body positron emission tomography (PET) scan, and vaginal ultrasound, were all negative for any visible solid-organ tumor such as ovarian teratoma or other underlying neoplasm.

Her hospital course was complicated by increasing agitation, psychotic episodes, seizures (clinical and subclinical, including non-convulsive status epilepticus), dystonia, and autonomic instability with paroxysmal sympathetic hyperactivity, leading to subsequent intubation and management in the ICU on day 13.

In total, the patient received first-line therapy consisting of methylprednisolone (1,000 mg IV for 5 days), IVIg (two rounds of 2 g/kg over 5 days each), and PLEX (two rounds for 5 days), followed by rituximab at 375 mg/m^2^ administered weekly (Figure 1). Given that PLEX can potentially negate the effects of rituximab, a second dose of rituximab was given after the completion of PLEX and following a discussion with hematology–oncology and neuroimmunology specialists who determined that a second round of rituximab was deemed clinically reasonable. Cyclophosphamide was considered but, given the potential gonadal toxicity and the patient's young age and future child-bearing potential, the mother requested an alternate immunotherapy. In consultation with various specialists including hematology and pharmacy and with preliminary reports of off-label bortezomib use for similar refractory cases (11–14), we opted to treat the patient with this drug.

Bortezomib was administered as four doses of 1.3 mg/m^2^ subcutaneously on hospital days 40, 43, 47, and 50. After the initiation of bortezomib, the patient showed a remarkable recovery with no drug tolerability issues, allowing weaning of sedation, and tracheostomy reversal on day 55 and subsequent transfer out of the ICU. She was discharged on hospital day 70 with oral prednisone taper and periodic outpatient IVIg infusions. She continued to show cognitive improvement at a follow-up appointment 5 weeks after discharge, with a Montreal Cognitive Assessment (MOCA) of 20/30. At follow-up 3 months later, CSF anti-NMDA titer levels had fallen to 1:64 and she self-reported feeling normal. She had a MOCA of 26/30 and had started online college.

## Discussion

The treatment of refractory anti-NMDA receptor encephalitis represents a clinical challenge as it is often associated with long-term intensive care and significant morbidity (9, 13, 14, 16). Here we describe a patient with a severe course of anti-NMDA receptor encephalitis without immediate response to first- and second-line treatment regimens of steroids, IVIG, plasma exchange, and rituximab. She was treated with bortezomib therapy as a novel second-line approach which resulted in dramatic and continued recovery. To the best of our knowledge, the case presented here represents the shortest hospitalization-to-bortezomib treatment timeline (42 days), and we believe that this is reflected in the patient's outcome with complete independence within a short timeframe.

Currently, patients typically receive treatment with cyclophosphamide after failing to respond to rituximab; however, anti-NMDA receptor encephalitis commonly affects young women and the potential toxicity of the drug, including consequences on fertility, needs to be taken into consideration (17). Cyclophosphamide was considered for this patient but, given her young age and the potential gonadal–ovarian toxic effects on long-term child-bearing potential, the patient's mother elected to use bortezomib instead of cyclophosphamide. Our multidisciplinary group including neurology, hematology, and critical care physicians and pharmacy consultants felt that this was a less toxic option for future child-bearing potential considerations in an 18-year-old female.

Prior case reports and retrospective case series (summarized in Table 1) have described a therapeutic response to bortezomib; however, without appropriate controls, the delayed effect of prior treatments cannot be ruled out. In one of the largest case series of five patients by Scheibe et al. (13), severely affected patients who demonstrated a delayed response or resistance to standard first- and second-line immunotherapy were administered one to six cycles of bortezomib. A partial NMDA receptor titer decline was observed in four out of the five patients, accompanied by clinical improvement and an acceptable safety profile for bortezomib. This was followed with a report by Sveinsson et al. (14) who described a severe case of treatment refractory anti-NMDA receptor encephalitis in a young woman. The patient received repeated doses of bortezomib at 147 days after hospital admission and invasive ventilation following intractable epileptic seizures. Remission was achieved after 204 days in intensive care and improvements in cognitive function were still ongoing 2 years after the disease onset. Further evidence to a specific effect of bortezomib is demonstrated by Schroeder et al. (15), with video documentation of the immediate visual improvement following bortezomib administration. In previously reported case studies, the patients receive heterogeneous first- and second-line pretreatment, and bortezomib was administered at variable points in disease progression.

**Table 1:** Literature review of NMDA encephalitis and refractory cases with bortezomib.

	**Age, gender, ethnicity**	**Time to diagnosis**	**Initial anti-NMDA serum/CSF titers**	**Clinical phenotype**	**Immunotherapy use and time to bortezomib treatment**	**References**
1	Early 30 s, female, African descent	Not reported	1:1,000 (serum) at ~2 months	Acute agitation, hallucinations, catatonia, autonomic instability, tetraparesis	Steroids, PLEX, IVIg, rituximab, cyclophosphamide, bortezomib (~8 months). Resolution at ~11 months.	(12)
2	Early 20s, female, Caucasian	Not reported	1:10 (serum) and 1:10 (CSF) at ~5 months	Behavioral changes, hallucinations, gait ataxia, central hypoventilation. Relapse 20 months later—gait ataxia, confusion, hallucinations, sexual disinhibition	PLEX, rituximab, steroids, IVIg, bortezomib (~29 months after initial onset)	
3	22, female	Not reported	Not detectable in serum and 1:10 (CSF)	Catatonia, psychosis, agitation, autonomic dysfunction, seizures	Steroids, PLEX, rituximab, cyclophosphamide, bortezomib (~7.5 months)	(13)
4	28, female	Not reported	1:100 (serum) and 1:3.2 (CSF)	Anxiety, panic attacks, aggression, hypersomnia, seizures	PLEX, IVIg, rituximab, cyclophosphamide, bortezomib (~68 months)	
5	19, female	Not reported	1:320 (serum) and 1:32 (CSF)	Psychosis, cardia arrest, seizures	Steroids, PLEX, rituximab, bortezomib (~6 months)	
6	22, female	Not reported	1:10,000 (serum) and 1:320 (CSF	Psychosis, dissociative behavior, chorea, hyperkinesia, tachycardia, seizures	Steroids, PLEX, IVIg, rituximab, bortezomib (~3 months)	
7	61, male	Not reported	1:320 (serum) and 1:100 (CSF)	Vegetative state, seizures	Steroids, PLEX, IVIg, rituximab, bortezomib (~17 months)	
8	26, female, Southeast Asian	1 month	1:2,500 (CSF) at diagnosis	Neuropsychiatric, aggression, hallucinations, insomnia, seizures	Steroids, rituximab, PLEX, IVIg, cyclophosphamide, tocilizumab, bortezomib (day 147). Discharged to rehabilitation clinic at day 313	(14)
9	22, female, Caucasian	Within first days of hospital admission	1:400 (CSF) at diagnosis	Subacute psychosis, seizures, motor stereotypies	Steroids, IVIg, PLEX, rituximab, bortezomib (3 months). Discharged to rehabilitation clinic at ~25–30 days later	(15)
10	20, female	First-line therapy initiated at 15 days	1:640 (serum) at admission	Vegetative state, motor stereotypies, autonomic instability, central hypoventilation, seizures	Steroids, IVIg, PLEX, rituximab, tocilizumab, bortezomib (~9 months)	(16)
11	28, female	First-line therapy initiated at 7 days	1:1,280 (serum) and 1:640 (CSF) at admission	Vegetative state, motor stereotypies, central hypoventilation, sympathetic paroxysmal hyperactivity, seizures	Steroids, IVIg, rituximab, cyclophosphamide, tocilizumab, IL2, bortezomib (~12 months)	
12	58, male	First line therapy initiated at 70 days	1:160 (serum) and 1:160 (CSF) at admission	Vegetative state, motor stereotypies, rigidity, seizures	Steroids, IVIg, rituximab, tocilizumab, bortezomib (~5 months)	
13	17, female	First line therapy initiated at 11 days	1:320 (CSF) at admission	Vegetative state, motor stereotypies, rigidity, sympathetic paroxysmal hyperactivity, seizures	Steroids, IVIg, rituximab, tocilizumab, bortezomib (~5 months)	
14	51, male	First line therapy initiated at 9 days	Not checked	Vegetative state, motor stereotypies, seizures	Steroids, IVIg, PLEX, rituximab, tocilizumab, bortezomib (~5 months)	
15	35, female	Within first days of hospital admission	Positive in CSF and serum	Personality disorder, emotional lability, amnesia, seizures, paranoia, hallucinations	Steroids, IVIg, PLEX, rituximab, cyclophosphamide, bortezomib (3 cycles; ~357 days)	(11)
16	49, female	Within first days of hospital admission	1:250 (CSF) and ~1:750 (serum)	Headache, myalgia, behavioral changes, seizures, involuntary movements	PLEX, IVIg, cyclophosphamide, rituximab, bortezomib (~11 months)	
17	18, female, African American	6 days	1:128 (CSF) at day 6	Severe agitation, seizures, dystonia	Steroids, IVIg, PLEX, rituximab, bortezomib (day 40). Hospital discharge on day 70	Current case

*NMDA, N-methyl-D-aspartate; CSF, cerebrospinal fluid; PLEX, plasma exchange; IVIg, intravenous immunoglobulin*.

In contrast, in a five-patient case series with historical controls, Shin et al. (16) report minimal improvements in clinical symptoms with no improvement in global modified Rankin Scale score following a bortezomib treatment of patients with severe anti-NMDA receptor encephalitis. Moreover, the study population demonstrated a comparable disease course to the historical control group. However, they conclude that this result may reflect the delay in treatment from disease onset to the administration of bortezomib (median: 5 months, range: 5–12 months). Perhaps the positive outcome of our illustrative case is partially reliant on the early timing of bortezomib administration. It could be that early administration of bortezomib allows for targeting of plasma cells prior to their crossing of the blood–brain barrier as bortezomib has poor central nervous system penetrance (18). Particularly in the case of a refractory autoimmune disease, it is possible that the antibody response is mediated by long-lived plasma cells. These cells do not respond to traditional immunosuppressive therapies such as B-cell depletion regimens as they do not express the cell surface CD20 antigen targeted by rituximab but instead may be vulnerable to targeting by proteasome inhibitors such as bortezomib (19, 20). In the absence of randomized, controlled trials to measure the efficacy against another standard immunotherapy, however, we cannot determine whether bortezomib is more efficacious than other therapeutic options.

Anti-NMDA receptor encephalitis can be heterogeneous and prognostic markers are lacking. Recently, a score to predict 1-year functional status has been published; however, the timing of escalation of therapy and the initial treatment strategy remain unclear (21). Sveinsson et al. (14) documented the CSF profiles of tissue injury biomarkers that correlate to disease staging. Their data demonstrated that early disease impacts the synaptic and dendritic processes that progress to neuroaxonal degeneration as the disease progresses clinically. Encouragingly, MRI imaging in long-term follow-ups of patients with anti-NMDA receptor encephalitis demonstrates that the frontotemporal atrophy occurring with the disease is potentially reversible (14, 22). Moreover, we report no side effects of this treatment, in contrast to other reported cases which can include peripheral neuropathy, gastrointestinal symptoms, and thrombocytopenia among others (13, 23).

## Conclusions

This case report and the previously published research suggest that bortezomib is a promising therapy for anti-NMDA receptor encephalitis. Our case represents the shortest hospitalization-to-bortezomib treatment timeline, and we believe that this is reflected in the patient's outcome with complete independence within a short timeframe. However, without prospective randomized trials, the therapeutic benefits of this therapy remain uncertain. Furthermore, the publication bias of favorable case reports can lead to optimistic conclusions. Nonetheless, we conclude that the clinical benefit of bortezomib and its treatment regimen warrant further validation in future randomized clinical trials and are required to guide clinical practice.

## Data Availability Statement

The datasets generated for this study are available on request to the corresponding author.

## Ethics Statement

Written informed consent was obtained from the individual for the publication of any potentially identifiable images or data included in this article.

## Author Contributions

MT designed and conceptualized the study, performed the literature review, and drafted the manuscript. TB and AS acquired the data and drafted the manuscript. JS, AL-C, and WF interpreted the data. All the authors revised the manuscript for intellectual content and approved the final version.

### Conflict of Interest

The authors declare that the research was conducted in the absence of any commercial or financial relationships that could be construed as a potential conflict of interest.
